# Phospholipase A1 Member A Deficiency Alleviates Mannan-Induced Psoriatic Arthritis in Mice Model

**DOI:** 10.3390/ijms23158559

**Published:** 2022-08-02

**Authors:** Yang Zhao, Fawzi Aoudjit, Sylvain G. Bourgoin

**Affiliations:** 1 Centre de Recherche du CHU de Québec-Université Laval, Université Laval, Quebec, QC G1V 4G2, Canada; yang.zhao@crchudequebec.ulaval.ca; 2 Centre ARThrite de l’Université Laval, Université Laval, Quebec, QC G1V 4G2, Canada; 3 Département de Microbiologie-Infectiologie et d’Immunologie, Université Laval, Quebec, QC G1V 0A6, Canada; fawzi.aoudjit@crchudequebec.ulaval.ca

**Keywords:** phospholipase A1A, mannan, cytokines, lymphocytes, IL-17, bone erosions, inflammation

## Abstract

Synovial fluids from rheumatoid and psoriatic arthritis patients have high levels of PLA1A. The current study was to understand PLA1A functions in the pathophysiology of rheumatic diseases. We generated Pla1a^−/−^ mice to assess their phenotype and the impact of PLA1A deficiency on the development of mannan-induced psoriatic arthritis (MIP). Mice were evaluated routinely for the induced symptoms. On the day of sacrifice, blood samples were collected for hematology analysis and prepared for plasma. Livers were collected. Lymph node immune cells were analyzed using flow cytometry. We performed μCT scans of hind paws from naïve and mannan-induced female mice. Cytokines/chemokines were quantified using Luminex in hind paw tissues and plasma of female mice. Pla1a^−/−^ mice showed a slight increase in circulating and lymph node lymphocytes. CD4^+^ T cells contributed most to this increase in lymph nodes (*p* = 0.023). In the MIP model, the lymph node ratios of CD3^+^ to CD19^+^ and CD4^+^ to CD8^+^ were higher in Pla1a^−/−^ mice. Pla1a^−/−^ mice were less susceptible to MIP (*p* < 0.001) and showed reduced bone erosions. Pla1a^−/−^ mice also showed reduced IL-17, KC, IP-10, MIP-1β, LIF, and VEGF in hind paw tissues as compared to WT mice (*p* < 0.05). These findings indicated that PLA1A deficiency protected from the development of the MIP disease. The data suggested that PLA1A could contribute to MIP through increased activation of lymphocytes, possibly those producing IL-17.

## 1. Introduction

Phospholipase A1 member A (PLA1A) specifically hydrolyzes sn-1 fatty acid of phosphatidylserine (PS) or 1-acyl-lysophosphatidylserine (1-acyl-lysoPS) [1]. PLA1A could play a proinflammatory role via the production of lysoPS and activation of lysoPS receptors expressed in myeloid cells. Increased PLA1A plasma/serum levels were associated with various autoimmune disorders, such as systemic lupus erythematosus [2] and early diagnosed rheumatic arthritic (RA) [3]. PLA1A levels in synovial fluid from RA and psoriatic arthritis (PsA) patients were elevated [3]. PLA1A added to a culture of human fibroblast-like synoviocytes (FLSs) contributed to local lysophosphatidic acid (LPA) and IL-8 synthesis through activation of the LPA receptors [3]. 

PsA is a chronic autoimmune disease characterized by synovial inflammation and synovial immune cell infiltration, which resembles RA. The functional impairment and radiological damage happen early in the disease course [4]. The Th17/IL-23 axis plays a role in the immunopathology of PsA [5]. IL-17 can regulate synovial inflammation and joint destruction through FLSs, osteoblasts, and chondrocytes activation [5], thereby maintaining disease chronicity. Other proinflammatory cytokines, such as TNF-α, IL-1β, IFN-γ, IL-6, IL-10, IL-12, IL-15, and various co-stimulatory molecules are expressed in PsA synovial tissues and are critical factors in disease progression [6]. 

Mannan is a natural ligand for mannose receptors and is known to trigger IL-17 pathways in the host [7]. The perception of mannan by innate immune cells is complex and cell-type-specific [8]. Mannan was used to induce arthritis in various mice strains [7,8,9,10,11,12]. Injection of mannan in SKG mice resulted in about 4-week-earlier and more severe arthritis when compared to injection of zymosan A [9]. The mannan-induced psoriatic arthritis (MIP) model described in 2014 showed that a single intraperitoneal injection of mannan induced acute inflammation resembling human psoriasis (Ps)- and PsA-like disease, and repeated injections induced relapsing disease [7]. The development of MIP disease is likely through the activation of macrophages or neutrophils expressing the mannose receptor [10]. Macrophages might be one of the cell types responsible for the enhanced inflammation in the MIP model [11]. High serum and local TNF-α may contribute to the development of the MIP disease [11]. TNF-α produced by macrophages can trigger the activation of γδT cells and the production of IL-17A [7]. Increased IL-17A contributed to the recruitment of neutrophils to the joints and skin [7]. The percentage of IL-17A-producing CD4^+^ T cells increased, and neutralization of IL-17 alleviated exacerbation of MIP in LACC1-deficient mice [11]. 

To better understand the role of PLA1A in inflammation and gain insight into how PLA1A contributes to the pathogenesis of autoimmune diseases, we characterized the Pla1a^−/−^ mice and assessed the implication of Pla1a in the development of MIP. Our results suggested that PLA1A deficiency slightly increased the CD3^+^CD4^+^ lymphocytes in lymph nodes. Pla1a^−/−^ mice were less susceptible to MIP. Reduction of tissue cytokine/chemokine levels, including IL-17, may alleviate inflammation and bone erosions. 

## 2. Results

### 2.1. Phenotypes of Pla1a^−/−^ Mice

**Figure 1:**
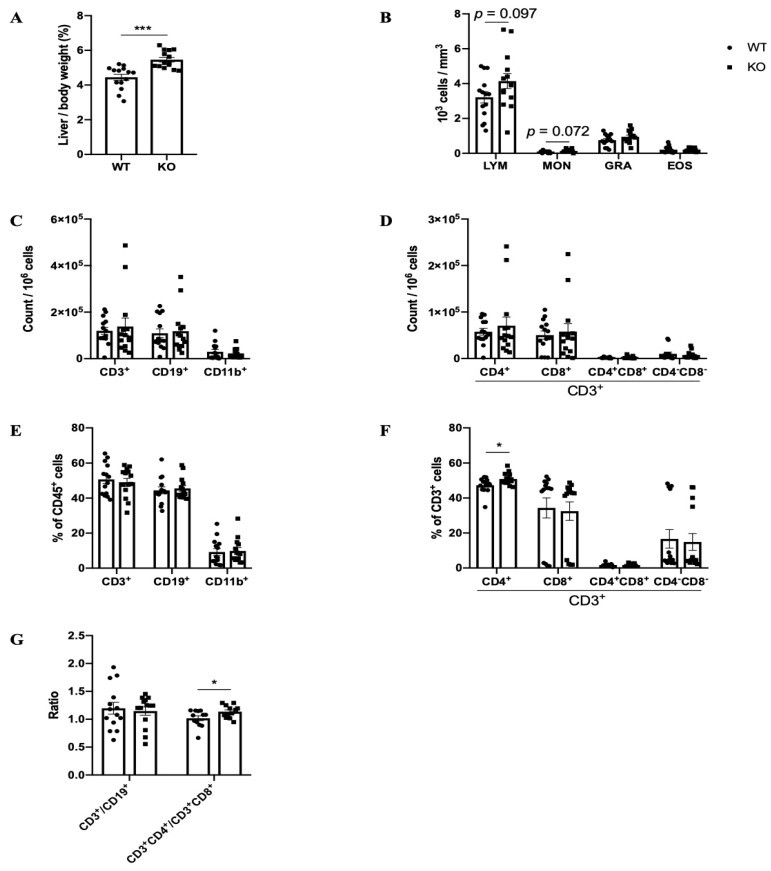
**Phenotype of Pla1a^−^^/^^−^ mice.** Age- and sex-matched mice (*n* = 14 per group, 7 males and 7 females) were euthanized to analyze the phenotype. (**A**) Liver-to-body weights ratio. (**B**) White blood cell counts in the blood. (**C**,**D**) Immune cell counts in the lymph node. (**E**,**F**) Percentage of immune cell populations in the lymph nodes quantified by flow cytometry. (**G**) Ratio of assigned cell populations. LYM, lymphocytes; MON, monocytes; GRA, granulocytes; EOS, eosinophils. *, *p* < 0.05; ***, *p* < 0.001; unnoted, *p* > 0.1.

Since we observed no differences between males and females, data of the two sexes were mixed in each group. Pla1a^−/−^ mice were slightly smaller but had a bigger liver (1.49 ± 0.08 g) compared to WT control (1.30 ± 0.06 g), as highlighted by the increased body to liver weight ratio (Figure 1A). There was a tendency for enhanced circulating levels of white blood cells (WBC) in Pla1a^−/−^ (5.23 ± 0.53 10^3^ cells/mm^3^) in comparison to WT mice (4.06 ± 0.40 × 10^3^ cells/mm^3^).

The cell counts of blood lymphocytes, monocytes, and granulocytes were not statistically different between WT and Pla1a^−/−^ mice (Figure 1B). The percentages of each type of WBC were not altered (data not shown). PLA1A deficiency had no significant impact on the levels of CD3^+^, CD19^+^, and CD11b^+^ cells in lymph nodes (Figure 1C–E). Among the CD3^+^ cells, the percentage of CD3^+^CD4^+^ cells and the CD3^+^CD4^+^ to CD3^+^CD8^+^ ratio increased in Pla1a^−/−^ mice (Figure 1F,G).

### 2.2. The Mouse Model of MIP

**Figure 2:**
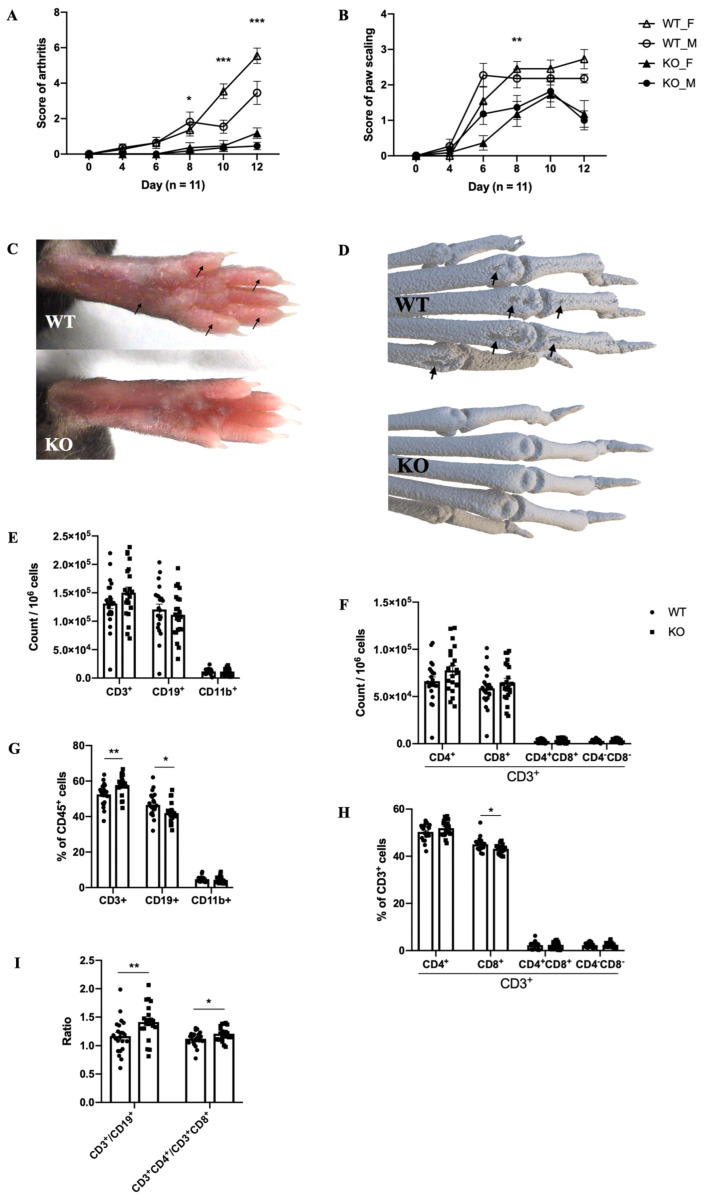
**Reduced susceptibility of Pla1a^−^^/^^−^ mice to MIP disease.** Age- and sex-matched mice were i.p. injected with 20 mg mannan on days 0, 4, and 8. Mice were evaluated on day 0 and every two days after day 4. Mice were euthanized on day 12. (**A**,**B**) Score of arthritis and paw scaling in males and females (*n* = 11 per group). (**C**,**D**) Representative images of sick hind paws and μCT scans (female) on day 12 after injection of mannan. Peeling, edema, and bone erosions were marked (arrows). The next figures combined data from males and females (*n* = 22 per group). (**E**,**F**) Quantification of immune cell populations in the lymph nodes. (**G**,**H**) Percentage of immune cell populations in the lymph nodes quantified by flow cytometry. (**I**) Ratio of assigned cell populations. For statistical comparative analyses in (**A**,**B**), we performed a multivariate analysis of the variance (MANOVA). *, *p* < 0.05; **, *p* < 0.01; ***, *p* < 0.001; unnoted, *p* > 0.1.

Pla1a^−/−^ mice developed less severe MIP symptoms when compared to WT mice. The incidence of disease in each group was 10/11 (male WT), 11/11 (female WT), 4/11 (male KO), and 8/11 (female KO), respectively. The score of arthritis was lower in Pla1a^−/−^ mice. Female WT developed more severe arthritis on day 10 and 12 (*p* < 0.001) when compared to males (Figure 2A). 

Compared to WT mice, Pla1a^−/−^ mice also showed reduced paw scaling (Figure 2B). Induced arthritis started in the hind paws after the second injection and reached the maximum severity level on day 12. Symptoms in front paws were rare as only a few WT mice (4/11 of males, 5/11 of females) and one Pla1a^−/−^ female developed very mild swelling and redness (score 1). The affected joints had classical signs of PsA, such as redness, swelling, and visible peeling of the skin (Figure 2C). PLA1A deficiency protected mice from bone erosions observable around the distal metacarpophalangeal joints (Figure 2D).

As for quantification of immune cells, differences between male and female were not significant (data not shown); therefore, the two sexes were mixed for the analyses. Compared to non-disease mice (Figure 1C), in the MIP model, there was a tendency for increased levels of CD3^+^ cells in the lymph nodes, particularly CD3^+^CD4^+^ (Figure 2E,F). However, the number of lymph modes CD3^+^CD4^−^CD8^−^ (Figure 2F) was lower in the MIP model compared to non-disease mice (Figure 1D). In the MIP model, the percentage of CD19^+^ and CD3^+^CD8^+^ decreased significantly in the lymph nodes of Pla1a^−/−^ mice (Figure 2G,H) and resulted in the increase of CD3^+^ to CD19^+^ and CD3^+^CD4^+^ to CD3^+^CD8^+^ ratio, respectively (Figure 2I). 

Except for IL-7, PLA1A deficiency had no significant impact on cytokine/chemokine plasma levels in non-disease mice (Table 1). In the MIP model, IL-7, IL-9, IL-17, KC, LIF, VEGF, MIP-1β, eotaxin, and IP-10 levels were significantly lower in paw tissues of Pla1a^−/−^ mice. On day 12 post-injection of mannan, the cytokine/chemokine levels in the plasma of Pla1a^−/−^ and WT mice were not significantly different.

## 3. Discussion

RA, SLE, and Graves’ disease patients show a high plasma level of PLA1A [1]. The underlying molecular mechanism by which PLA1A regulates the immune response is still not well understood. To address this issue, we investigated the effects of PLA1A deficiency in the mouse model of MIP, which is IL-17-driven [10]. PLA1A deficiency reduced susceptibility to induced arthritis and cutaneous inflammation in the MIP model. Reduced MIP symptoms were associated with a reduction of several proinflammatory chemokines/cytokines, including IL-17 in hind paw tissues of Pla1a^−/−^ mice. 

Pla1a^−/−^ mice were healthy and showed no particular phenotype up to 10 months of age. Careful analysis revealed that the Pla1a^−/−^ animals were slightly smaller, although with larger livers. PLA1A is highly expressed in the liver, which is a possible source of circulating PLA1A [1]. PLA1A deficiency may impact mouse metabolism and liver functions. Whether this contributes to protecting mice from arthritic and cutaneous inflammation remains to be investigated.

PLA1A might contribute to the development of arthritis in the MIP model by promoting IL-17-producing cells since PLA1A deficiency was associated with a lower level of IL-17. Innate immune cells can sense components of fungal cell walls such as mannan, a typical danger-associated molecular pattern, and initiate the MIP in mice [8]. Macrophage activation by mannan is known to induce the production of TNF-α and IL-1α, which trigger the local production of IL-17 by γδT cells [7] and ILC3 [10], respectively. γδT cells and ILC3 expansions could enhance the susceptibility of psoriasis in mice [12]. In this context, PLA1A deficiency might affect the mannan detection and the activation of immune cells (CD11b^+^ cells), subsequently leading to the impaired responses of IL-17-producing cells. 

Previous studies reported the increase and activation of CD8^+^ T cells [5,13] and conversion of CD4^+^ T cells into Th17 cells [14] in PsA. Human CD4^+^ T cells express a high level of PLA1A mRNA [3]. In addition, PLA1A can affect T cell activation through the generation of bioactive lysophospholipid lysoPS and activation of lysoPS receptors that are highly expressed by T cells [1]. In the current study, Pla1a^−/−^ mice injected with mannan showed lower proportions of B cells and CD8^+^ T cells. The decreased lysoPS level in Pla1a ^−/−^ mice might modulate CD4^+^ T cells activation and polarization towards Th17 through activation of lysoPS receptor GPR174 [1]. These alterations possibly contribute to the surprisingly low Pla1a^−/−^ mice susceptibility to mannan-induced psoriatic-like symptoms. Further studies should address whether PLA1A deficiency affects T cell trafficking, T cell activation, or a skewing towards proinflammatory phenotypes. 

Female mice developed more severe arthritis in response to mannan, as described previously in SKG mice [9]. Erosions detected in WT mice were not present in Pla1a^−/−^ mice injected with mannan. Joint erosions are the most frequent and early radiological features in PsA patients, which are associated with a poor prognosis [4]. The second and third metacarpals are mechanically vulnerable sites and are prone to erosions. KC [11] and IL-17A [7] contribute to bone erosion in the animal models. In PsA patients, TNF-α was detectable in synovial fluids and synovial lining cells [15], and IL-7 in the sera and synovial fluids [13]. TNF-α contributes to bone destruction by increasing the frequency of osteoclast precursors in the circulation and their migration to the inflamed synovium and subchondral bone, where osteoclast precursors meet RANKL [15]. Activated T cells and fibroblasts support osteoclastogenesis formation and aggressive bone resorption by producing RANKL, TNF-α, IL-6, IL-7, and MIP-1α in PsA patients [13]. In this MIP model, cytokines responsible for bone resorption, such as IL-6, IL-7, IL-17, KC, MIP-1α, and TNF-α, were reduced in Pla1a^−/−^ mice. VEGF, which contributes to angiogenesis in the PsA synovium, was significantly lower in Pla1a^−/−^ mice.

This study has several limitations. Firstly, limited immune cell populations were analyzed. Further characterization of the macrophage and lymphocyte subsets infiltrating the inflamed hind paws is needed. Secondly, we did not characterize the articular phenotype. A histological analysis would complete the clinical evaluation of this mouse model. Finally, the study does not discriminate between the intracellular and extracellular functions of PLA1A, and the mechanisms by which PLA1A contributes to MIP disease remain to be established. 

In summary, we provided novel information on the inflammatory networks regulated by PLA1A in vivo. We suggested that the reduced incidence and severity of MIP were mainly due to the modulation of immune cell polarization towards IL-17 and activation by PLA1A. Pla1a^−/−^ mice should allow a better understanding of the PLA1A functions in the etiology of systemic autoimmune rheumatic diseases.

## 4. Materials and Methods

### 4.1. Mice 

Applied StemCell Inc. (Milpitas, CA, USA) generated the Pla1a^−/−^ mice. All mice were bred in their respective genetic backgrounds (C57BL/6J). Genotyping of mice was performed using ear biopsies from mice of 4 weeks of age. The PLA1A-specific primers for genotyping were 5′-TCACTCGGCCCCACAACAGC-3′ (F), and 5′-GCCTCTTCATCTCCCCGTCATCT-3′ (R). Mice housing occurred in a pathogen-free animal facility. Mice were maintained under 12 h light–dark cycles and they received water/food ad libitum. Laval University Animal Protection Committee approved the experiments (protocol no. 17-157). The experiments used mice between 12 to 40 weeks of age. Mice were anesthetized with isoflurane to collect the blood samples by cardiac puncture. Hematology testing used blood samples (100 µL) in EDTA solution. Analysis used the scil Vet abc^+^ hematology analyzer (Scil Animal Care Company Inc., Barrie, ON, Canada). Following centrifugation of blood samples in the anticoagulant–citrate–dextrose solution, the collected plasma was stored at −80 °C. Lymph nodes were kept in iced PBS, immune cells were prepared and analyzed within 30 min. Livers were weighed and quick-frozen and kept at −80 °C for future analysis. Mice hind paws were removed 0.2 cm above the ankle and stored in 15 mL falcon tubes at −20 °C.

### 4.2. Model of MIP

The MIP model was induced in WT and Pla1a^−/−^ mice of 12 to 13 weeks of age following a widely used protocol [7]. All randomly assigned experimental groups consisted of littermate sex- and age-matched mice. Mice were i.p. injected with 20 mg mannan (Sigma Aldrich, Oakville, ON, Canada) in 200 µL phosphate-buffered saline (PBS) on days 0, 4, and 8. Mice were weighed and monitored for clinical signs of MIP on day 0 and every two days after day 4. Arthritic paw scores were as follows: 0, no swelling and redness; 1, mild swelling and/or redness, flexibility present; 2, moderate swelling and/or redness, flexibility present; 3, moderate swelling and/or redness, little flexibility; 4; severe swelling and redness, no flexibility. Paw scaling score was as follows: 0, no; 1, mild; 2, moderate; 3, severe scaling. All mice were blindly evaluated by two persons. Mice were euthanized on day 12 and samples were collected immediately after the sacrifice.

### 4.3. Immune Cell Sorting

Preparation of single immune cell suspensions of mice lymph nodes used a standard protocol. Briefly, lymph nodes were crushed with PBS using a 40 µm nylon cell strainer (BD Biosciences, ON, Canada). The single-cell suspensions were centrifuged and resuspended in iced PBS buffer and stained with fluorescent-conjugated antibodies (BD Biosciences, Mississauga, ON, Canada) for surface markers: marking viability, APC-Cy7 anti-CD45, V450 anti-CD11b, PerCPCy5.5 anti-CD19, APC anti-CD3, FITC anti-CD4, PE anti-CD8. Polybead microspheres of 15 µm diameter (Polyscience, Inc., Warrington, PA, USA) were added to each sample before flow cytometry analysis (BD LSR II flow cytometer, BD Biosciences, ON, CAN). Data analysis was compiled using FlowJo software (BD Biosciences, Mississauga, ON, Canada).

### 4.4. Luminex

Hind paw tissues were mixed in PBS buffer with a protease inhibitor cocktail (Millipore-Sigma, St. Louis, MO, USA) and sonicated on the ice at a low intensity for 30 s. The tissue lysates were centrifuged twice at 4000× *g* (5 min, 4 °C) and the supernatants were aliquoted and stored at −80 °C. Before being sent to analysis, all samples were centrifuged at 10,000× *g* (10 min at 4 °C). The proteins were quantified (BCA protein quantification kit, Thermo Fisher Scientific, Ottawa, ON, Canada) and diluted to adjust protein content in samples at the same concentration. Plasma samples were diluted twofold in PBS buffer and sent to Eve Technology (Calgary, AB, Canada) for Luminex quantification of cytokines/chemokines.

### 4.5. Micro-Computed Tomography (μCT) Image Acquisition

Hind paw scans used GE eXplore Locus Ultra micro-CT scanner with the help of Professor Marc-André Fortin. The 3D image reconstruction used Microview software (GE Healthcare Technologies, Milwaukee, WI, USA).

### 4.6. Statistics

Statistical analyses were performed using GraphPad Prism (GraphPad Software, San Diego, CA, USA). Unless otherwise stated, the mean comparison between two groups used a *t*-test. All results were the mean ± SEM. A *p* < 0.05 was considered statistically significant. 

## Figures and Tables

**Table 1:** **Cytokine/chemokine levels in paw lysates and plasma**.

	Paw Lysate (pg/mL, *n* = 5)			Paw Lysate (pg/mL, *n* = 5)			Plasma (pg/mL, *n* = 5)		
	Control Mice			MIP			MIP		
	WT	KO	*p* Value	WT	KO	*p* Value	WT	KO	*p* Value
**Eotaxin**	72.97 ± 8.85	106.22 ± 64.79	0.339	176.10 ± 27.21	132.03 ± 11.59	0.018	238.52 ± 39.65	288.77 ± 26.23	0.067
**G-CSF**	6.60 ± 0.56	9.78 ± 3.19	0.085	152.47 ± 62.87	73.30 ± 28.62	0.051	355.06 ± 66.26	295.83 ± 64.08	0.235
**GM-CSF**	11.60 ± 0.88	12.13 ± 1.75	0.602	21.74 ± 1.94	26.24 ± 8.30	0.322	32.60 ± 3.45	33.89 ± 2.69	0.573
**IFNγ**	4.11 ± 0.93	3.43 ± 0.90	0.321	2.36 ± 0.31	2.81 ± 0.44	0.140	11.57 ± 4.20	15.63 ± 8.90	0.433
**IL-1α**	1298.44 ± 206.51	1745.13 ± 567.59	0.177	1586.86 ± 302.61	2259.35 ± 546.18	0.063	128.78 ± 22.24	146.74 ± 26.17	0.326
**IL-1β**	9.66 ± 0.90	9.02 ± 1.21	0.417	23.04 ± 5.52	21.38 ± 7.29	0.725	12.78 ± 0.54	16.82 ± 10.01	0.443
**IL-2**	7.52 ± 2.80	5.35 ± 1.97	0.241	3.55 ± 0.23	3.39 ± 0.48	0.582	29.44 ± 8.83	29.93 ± 16.02	0.959
**IL-3**	1.85 ± 0.11	1.79 ± 0.14	0.552	8.69 ± 11.07	3.34 ± 2.41	0.372	3.11 ± 0.43	3.80 ± 1.62	0.439
**IL-4**	0.54 ± 0.13	0.50 ± 0.09	0.650	0.83 ± 0.58	0.64 ± 0.15	0.541	0.82 ± 0.11	1.09 ± 0.35	0.179
**IL-5**	3.43 ± 1.90	3.74 ± 2.06	0.828	2.13 ± 0.14	2.41 ± 0.79	0.500	7.04 ± 1.53	5.97 ± 1.27	0.311
**IL-6**	6.05 ± 0.36	5.58 ± 1.83	0.623	20.88 ± 5.07	14.67 ± 10.82	0.329	5.35 ± 2.48	5.66 ± 2.29	0.856
**IL-7**	7.92 ± 0.75	6.62 ± 0.62	0.028	7.00 ± 0.75	5.61 ± 0.50	0.015	20.22 ± 11.64	9.37 ± 1.32	0.101
**IL-9**	24.23 ± 1.19	22.58 ± 2.01	0.196	21.11 ± 1.02	19.35 ± 1.09	0.046	83.84 ± 30.47	69.96 ± 7.32	0.402
**IL-10**	26.15 ± 3.70	20.18 ± 7.17	0.177	19.86 ± 8.83	13.93 ± 4.41	0.264	25.12 ± 2.64	25.44 ± 6.10	0.925
**IL-12p40**	44.37 ± 4.56	34.16 ± 7.92	0.054	31.02 ± 6.35	29.32 ± 1.87	0.620	24.97 ± 2.85	27.10 ± 2.25	0.274
**IL-12p70**	16.47 ± 1.41	15.87 ± 1.19	0.532	26.65 ± 13.31	19.73 ± 4.60	0.354	36.88 ± 7.88	51.29 ± 9.54	0.048
**IL-13**	3.49 ± 1.49	3.18 ± 0.85	0.725	2.98 ± 0.81	2.11 ± 0.42	0.095	204.48 ± 37.72	217.22 ± 59.07	0.725
**IL-15**	40.84 ± 6.33	46.98 ± 3.24	0.123	41.18 ± 3.37	36.88 ± 3.62	0.120	228.26 ± 132.86	135.21 ± 13.93	0.201
**IL-17**	1.12 ± 0.17	1.12 ± 0.54	0.989	84.83 ± 33.40	26.87 ± 15.76	0.014	3.83 ± 1.04	3.70 ± 1.19	0.875
**IP-10**	2.41 ± 0.44	2.75 ± 0.75	0.456	7.62 ± 1.20	4.33 ± 1.04	0.003	70.23 ± 25.89	51.20 ± 10.47	0.210
**KC**	29.40 ± 4.74	35.14 ± 13.30	0.440	136.06 ± 20.35	82.05 ± 29.22	0.016	25.48 ± 6.42	23.39 ± 11.65	0.761
**LIF**	9.62 ± 1.32	11.01 ± 0.61	0.093	25.66 ± 5.20	14.76 ± 1.98	0.004	8.50 ± 10.72	2.49 ± 0.80	0.296
**MCP-1**	78.25 ± 16.37	113.02 ± 49.95	0.222	229.29 ± 129.40	139.90 ± 76.34	0.268	45.41 ± 5.00	62.20 ± 41.74	0.448
**M-CSF**	21.42 ± 4.50	20.41 ± 3.21	0.724	16.24 ± 2.10	15.91 ± 3.43	0.872	38.51 ± 2.67	36.84 ± 2.66	0.403
**MIG**	5.99 ± 3.50	6.83 ± 3.54	0.745	19.63 ± 8.05	12.96 ± 3.61	0.169	259.55 ± 35.17	607.34 ± 371.66	0.099
**MIP-1α**	27.90 ± 1.47	28.19 ± 1.88	0.810	70.12 ± 10.76	58.56 ± 12.08	0.191	136.01 ± 17.20	130.96 ± 27.26	0.762
**MIP-1β**	18.18 ± 4.50	17.24 ± 3.04	0.739	44.31 ± 6.53	32.14 ± 7.34	0.038	95.25 ± 10.97	101.15 ± 27.23	0.698
**MIP-2**	180.89 ± 11.94	167.39 ± 26.58	0.381	1049.48 ± 301.44	859.24 ± 304.88	0.401	733.26 ± 3.52	735.67 ± 2.42	0.292
**RANTES**	1.14 ± 0.15	1.22 ± 0.22	0.576	2.97 ± 1.24	2.21 ± 0.64	0.308	21.92 ± 3.13	26.81 ± 7.59	0.268
**TNF-α**	3.41 ± 0.30	3.40 ± 0.60	0.991	10.90 ± 2.52	9.16 ± 2.77	0.380	18.17 ± 1.28	19.83 ± 3.82	0.433
**VEGF**	21.07 ± 1.71	24.68 ± 5.73	0.261	75.54 ± 7.03	54.11 ± 13.11	0.020	1.48 ± 0.41	1.37 ± 0.20	0.658
**LIX**							110.40 ± 52.50	85.28 ± 65.33	0.565

## Data Availability

All data are included in the manuscript.
